# Simultaneous Comparison of Aqueous Humor and Serum Metabolic Profiles of Diabetic and Nondiabetic Patients Undergoing Cataract Surgery—A Targeted and Quantitative Metabolomics Study

**DOI:** 10.3390/ijms241612671

**Published:** 2023-08-11

**Authors:** Emil Tomasz Grochowski, Karolina Pietrowska, Adrian Godlewski, Wioleta Gosk, Angelika Buczynska, Malgorzata Wojnar, Joanna Konopinska, Adam Kretowski, Michal Ciborowski, Diana Anna Dmuchowska

**Affiliations:** 1 Department of Ophthalmology, Medical University of Bialystok, M. Sklodowskiej Curie 24a, 15-276 Bialystok, Poland; emil.tomasz.grochowski@gmail.com (E.T.G.); wojnargosia@o2.pl (M.W.); joanna.konopinska@umb.edu.pl (J.K.); 2 Metabolomics Laboratory, Clinical Research Center, Medical University of Bialystok, M. Sklodowskiej Curie 24a, 15-276 Bialystok, Poland; karolina.pietrowska@umb.edu.pl (K.P.); adrian.godlewski@umb.edu.pl (A.G.); wioleta.gosk@umb.edu.pl (W.G.); angelika.buczynska@umb.edu.pl (A.B.); adam.kretowski@umb.edu.pl (A.K.); 3 Department of Endocrinology, Diabetology and Internal Medicine, Medical University of Bialystok, M. Sklodowskiej Curie 24a, 15-276 Bialystok, Poland

**Keywords:** metabolomics, targeted metabolomics, diabetes, diabetic retinopathy, cataract, aqueous humor, mass spectrometry

## Abstract

The aim of this study was to compare the aqueous humor (AH) and serum concentrations of metabolites in diabetic (*n* = 36) and nondiabetic (*n* = 36) senior adults undergoing cataract surgery. Blood samples were collected before surgery and AH during surgery. Liquid chromatography coupled with tandem mass spectrometry (LC-MS/MS)-based targeted metabolomic and lipidomic analyses of samples were performed using the AbsoluteIDQ^®^ p180 kit. Out of 188 metabolites targeted by the kit, 41 and 133 were detected in >80% of AH and serum samples, respectively. Statistical analysis performed to indicate metabolites differentiating diabetic and nondiabetic patients showed 8 and 20 significant metabolites in AH and serum, respectively. Pathway analysis performed for significant metabolites revealed that galactose metabolism is mostly affected in the AH, while arginine biosynthesis is mostly affected in the serum. Among metabolites that differentiate diabetic and nondiabetic patients, arginine was the only metabolite common to both serum and AH samples, as well as the only one with a decreased concentration in both body fluids of diabetic patients. Concentrations of the rest were elevated in AH and lowered in serum. This may suggest different mechanisms of diabetes-related dysregulation of the local metabolism in the eye in comparison to systemic changes observed in the blood.

## 1. Introduction

In 2019, the prevalence of diabetes mellitus (DM) was estimated at 11.3% of the U.S. population; of these cases, 23% (or 3.4% of all U.S. adults) were undiagnosed [1]. Diabetic retinopathy (DR) is among the leading causes of vision loss and blindness worldwide [2]. Among patients with DM, the overall prevalence was 34.6% for any DR, 6.96% for proliferative DR, and 6.81% for diabetic macular edema [3].

Cataract, the opacification of the lens in the eye, affects visual acuity and is the leading cause of blindness worldwide [4]. Cataract prevention in diabetics is of great importance, not only because of a five-fold higher risk of cataract development for diabetics but also due to an increased risk of intra- and postsurgical complications [5].

Although much has been established concerning major risk factors for the progression of non-proliferative and proliferative DR on a systemic level (hyperglycemia, hypertension, etc.) [5], our understanding of the underlying pathophysiology at the local level [6,7] can still be improved, thus expanding the potential for more tailored prevention and treatment.

The aqueous humor (AH) fills the anterior and posterior chambers of the eye, supplies nutrients, and removes waste products from avascular tissues, including the lens. While the vitreous body is potentially a better tissue for the study of retinal pathology due to its proximity, in diabetic patients with early or preclinical stages of DR, who do not need vitrectomy, vitreous sampling may represent an unjustified procedure, while AH sampling proved to be more applicable, equally reliable, and meaningful [8]. Its volume in the human eye is approximately 0.3 mL. Collection of AH samples is possible only during surgery.

According to a recent review [8], only two untargeted metabolomics studies investigated differences in AH composition in patients with and without diabetes [9,10], while three additional studies were focused on patients with proliferative stage diabetic retinopathy [11,12,13]. More recently, the first metabolomics analysis of AH in diabetic macular edema [14] and the first targeted metabolomic study of AH in DM were published [15].

Although the number of metabolites usually detected by a targeted approach is much fewer compared to a nontargeted approach [16], quantitative targeted analyses allow for the comparison of data obtained at different times and in a multicenter manner [17].

In the present study, we measured the concentrations of AH and serum metabolites in samples collected from diabetic (with no signs of diabetic retinopathy) and nondiabetic adults undergoing cataract surgery. We excluded patients with any ocular pathologies to ascertain if this methodology could identify metabolites dysregulated at the earliest preclinical stages of diabetic retinopathy in the AH and serum of DM patients. We also aimed to obtain better insight into the relationship between metabolomic signatures characteristic of diabetes at the systemic and those at the local level, as this may help recognize potential targets for preventing disease presence and progression.

To our knowledge, this is the first targeted, quantitative metabolomics study of AH and serum from the same DM patients to simultaneously correlate the metabolomic signatures of both biological materials. The indisputable value of this research is the fact that this is the first metabolomics study of AH in which 72 patients are included, for a total of 144 analyzed samples, as opposed to previous studies with 30 to 50 samples [8,9,10,11,12,13,14,15].

## 2. Results

The most relevant clinical data are presented in Table 1. Some differences in patient characteristics between groups merit additional commentary. Both groups were similar in terms of age, sex, BMI, and axial length of the eye, while the diabetes mellitus group was characterized by significantly higher concentrations of fasting glucose and lower levels of C-peptide. This indirectly confirms the correct diagnosis of patients and, therefore, group assignment. None of the patients showed any signs of diabetic retinopathy. For further details, please see Supplementary File S1.

### 2.1. Univariate Analysis

In the case of serum samples, in total, 133 metabolites fulfilled the inclusion criteria, and concentrations of 20 of them were significantly lower in the diabetic group, as shown in Table 2.

In the case of AH samples, in total, 41 metabolites fulfilled the inclusion criteria, and concentrations of eight of them were significantly elevated in the diabetic group, with the notable exception of arginine, which was decreased, as seen in Table 3.

### 2.2. Correlation between Concentrations of Metabolites in AH and Serum

**Figure 1:**
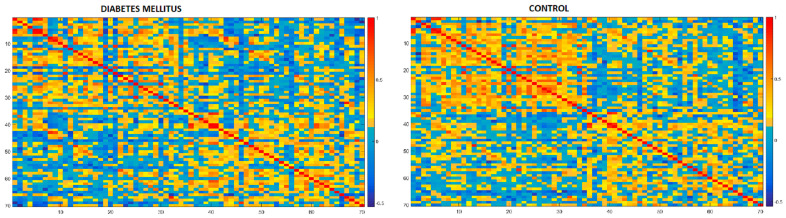
Correlation heatmap.

In total, 35 metabolites fulfilling the inclusion criteria were detected in serum and AH samples and were correlated (Figure 1). All correlated metabolites are listed in Table 4. For most of the metabolites, we observed a positive or no correlation between their concentrations in serum and AH in diabetic and nondiabetic patients. However, there are some metabolites for which the correlation between serum and AH was observed only for diabetic or nondiabetic patients. Especially glutamate is of special interest, as its neurotoxic, pro-inflammatory role in preclinical stages of diabetic retinopathy was investigated in animal models [18].

### 2.3. Multivariate Analysis

**Figure 2:**
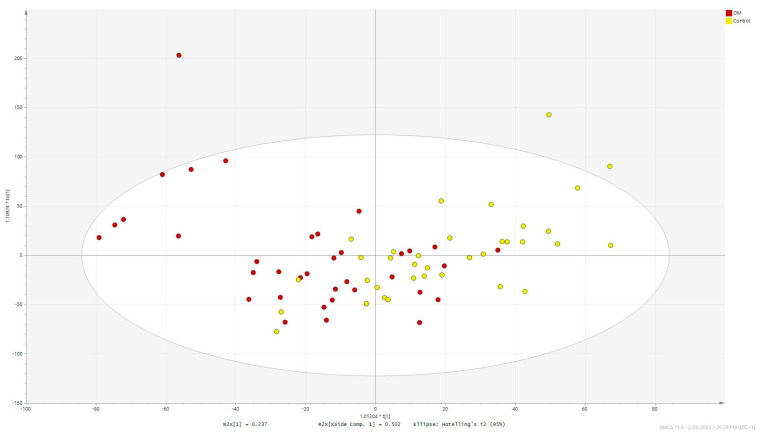
OPLS−DA score scatter plot obtained for serum data.

Orthogonal partial least squares discriminant analysis (OPLS-DA) was performed for metabolites significant in serum (Figure 2, R2Ycum = 0.357, Q2cum = 0.25).

**Figure 3:**
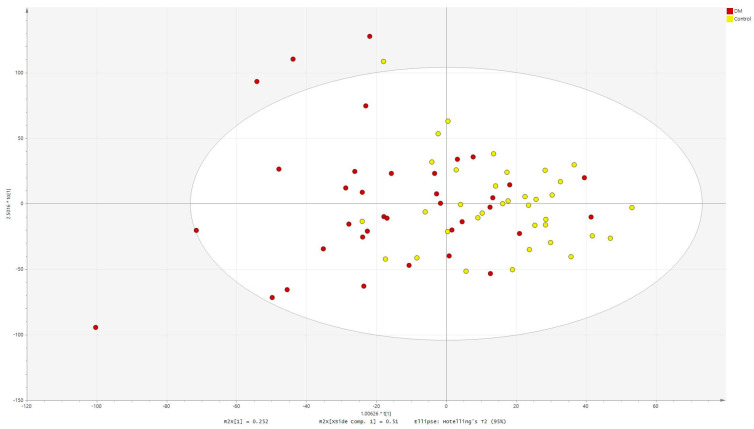
OPLS−DA score scatter plot obtained for aqueous humor data.

Orthogonal partial least squares discriminant analysis (OPLS-DA) was performed for metabolites significant in AH (Figure 3, R2Ycum = 0.293, Q2cum = 0.0623).

### 2.4. Pathway Analysis

Even though multiple metabolites were assigned to several pathways, the impact value was zero or a small value. Meanwhile, only one metabolite was assigned to most pathways. Nevertheless, we present these data for future reference.

**Figure 4:**
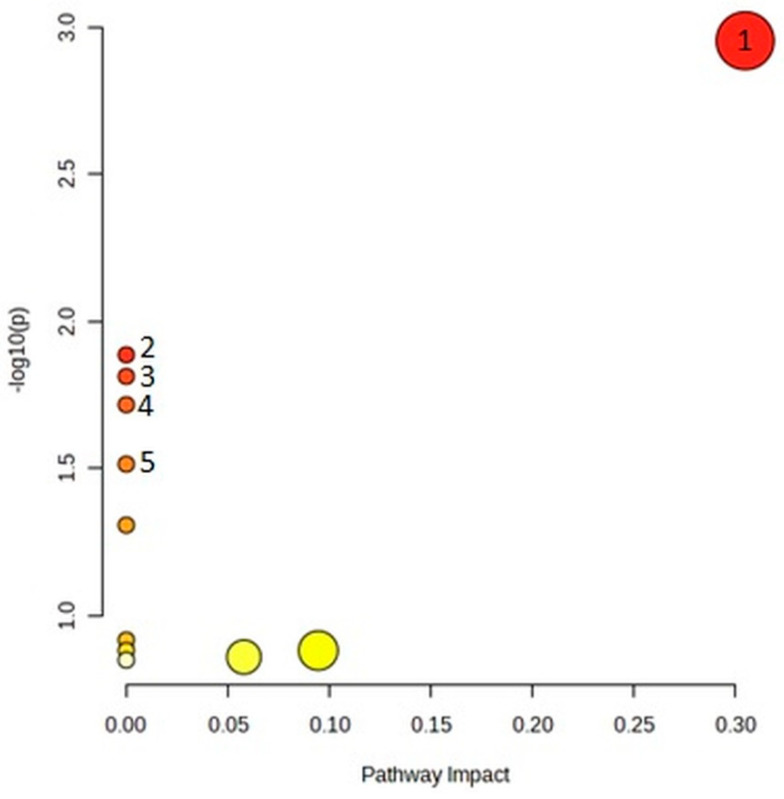
Pathway analysis for serum metabolites significantly discriminating between diabetic and nondiabetic patients. Five most significant pathways are numbered the same as in the corresponding table (Table 5).

From the list of 20 metabolites that significantly differentiated serum samples, in topology analysis, the arginine biosynthesis pathway was particularly significant (*p* = 0.0011, impact = 0.3), as seen in Figure 4 and Table 5.

**Figure 5:**
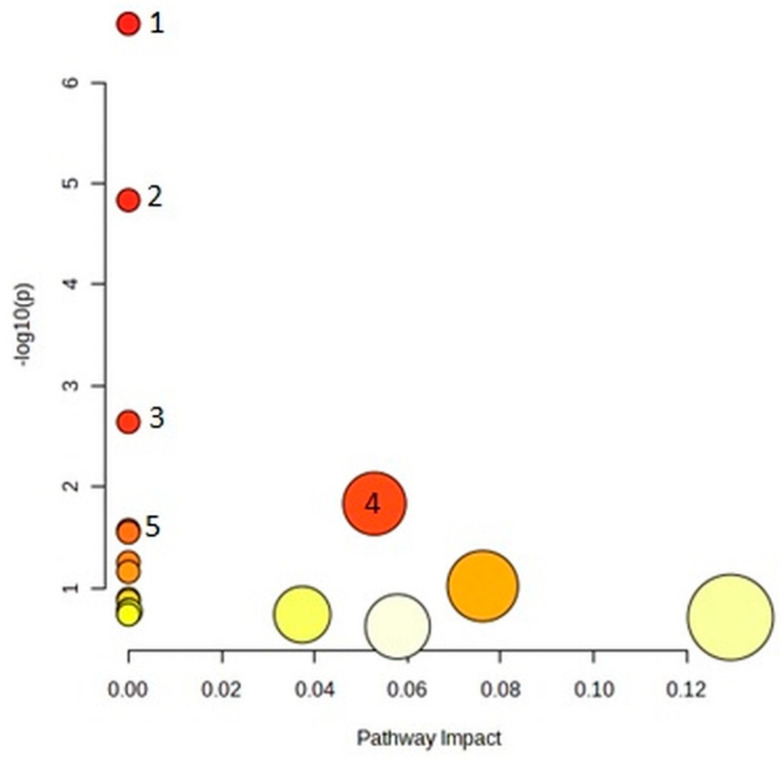
Pathway analysis for AH metabolites significantly discriminating between diabetic and nondiabetic patients. Five most significant pathways are numbered the same as in the corresponding table (Table 6).

From the list of eight metabolites that significantly differentiated AH samples in topology analysis, the galactose metabolism pathway was particularly significant (*p* = 0.015, impact = 0.052), as seen in Figure 5 and Table 6.

## 3. Discussion

Our goal was to compare the metabolomic profiles of AH and serum collected from diabetic and nondiabetic patients. Both studied groups were matched in terms of sex, age, and BMI. Moreover, diabetic patients were recently diagnosed and relatively well treated since they had not yet developed any signs of diabetic retinopathy.

As expected, due to the blood–aqueous barrier, more metabolites were identified in serum than in AH.

Half of the metabolites differentiating both groups in serum were various glycerophospholipids but also included acylcarnitines, amino acids, and sphingolipids. Their average concentrations were lower in the serum of diabetic patients.

Metabolites differentiating both groups in AH were mostly amino acids but also included propionyl carnitine and sugars. Arginine concentration was lower, while the concentrations of all the other metabolites were elevated in AH of diabetic patients. Of note, the average concentration of sugars was almost twice higher in the AH of diabetic patients, while in serum, significant differences were not observed. On the other hand, glucose measured in plasma using a hexokinase enzymatic assay was significantly higher in diabetic patients but to a smaller degree.

Arginine was the only metabolite differentiating groups simultaneously in both tested body fluids. Different dysregulation on systemic and local levels might account for this observation [19]. Disruption of the blood–aqueous humor barrier, associated with local inflammation preceding the development of clinically discernible diabetic retinopathy, is another possible factor [20].

Lower arginine concentration in AH of DM patients might also be associated with its antioxidant and anti-inflammatory properties [21]. Nitric oxide (NO) is synthesized from arginine by NO synthase (endothelial or induced). In turn, NO regulates the metabolism of glucose, fatty acids, and amino acids in mammals. A state of imbalance can occur in case of increased oxidative stress or if cellular transport of arginine is inhibited. Nitric oxide (NO) signaling has been studied in the eye, including in the pathophysiology of some eye diseases [22]. However, full coverage of this topic exceeds the framework of this paper.

Glutamate levels in AH and serum were positively correlated with each other only in the DM group. Glutamate metabolism was reportedly disturbed in AH of patients with proliferative diabetic retinopathy [13]. Considering its neurotoxic, pro-inflammatory role in preclinical stages of diabetic retinopathy was previously investigated only on animal models [18], our findings provide a new context for studies on human subjects.

The level of acylcarnitines is also known to increase in the blood of prediabetics [23]. In our previous study, butyrylcarnitine and decenoylcarnitine were found to be negatively correlated with AH glucose levels [9].

Comparison to the preceding research is necessary to facilitate the interpretation of our current findings. In our previous, untargeted, LC-MS metabolomics study of only the AH of diabetic and nondiabetic patients [9], arginine was also found to be significantly different. Likewise, on account of including serum in our recent research, metabolic pathways of arginine biosynthesis for serum were observed to be significantly impacted. For comparison, in our previous interstudy pathway analysis of serum in myopia research, arginine biosynthesis was not significantly altered (*p* = 0.7). However, the same analysis for AH identified arginine biosynthesis as the most impactful (*p* = 0.000028, impact = 0.3) [24]. In the current study, we were able to validate some of our previous results on a new group of patients with diabetes.

In the recently published paper, Lillo et al. also used the AbsoluteIDQ^®^ p180 kit to compare AH metabolic profiles of diabetic and nondiabetic patients [15]. However, they studied only 7 DM patients, and the study groups were not matched. DM patients were significantly older than the control group (72 (range 65–76) vs. 56 (range 24–76)). The authors do not report the mean value. Moreover, “healthy” control samples were presumably collected exclusively from myopic and hyperopic patients, indicated by quite extreme ranges of the axial length of the eye (21.25 mm–27.29 mm). These ocular disorders significantly disrupt the local metabolome [24,25] and greatly increase the risk of other ocular pathologies, the statuses of which are unclear, with the exception of diabetic retinopathy. Lack of information on this matter might be omitted by non-ophthalmologists; nevertheless, this should be considered a major confounding factor. We can partially corroborate their results as they, similarly to us, observed an increase in sugars, alanine, lysine, and propionylcarnitine in the AH of DM patients.

Yao et al. also compared the metabolic composition of AH collected from DM and controls, obtaining 20 significant metabolic features, of which almost half remained unidentified [10]. The problem of identification is clearly omitted in targeted studies like the one presented here. Nonetheless, none of the identified metabolites coincide with these significant in our work. However, such discrepancies can be explained by the use of a different analytical method (GC-MS). Different analytical methods used in metabolomics studies are suitable for the measurement of metabolites with various properties and belonging to distinct classes, therefore, providing complementary results [26]. Consequently, a multiplatform approach would be ideal to deeply explore differences in metabolome [6,7,8].

Kunikata et al. intended to profile 31 low-molecular-weight reactive polysulfides and their related molecular species in the aqueous and vitreous humor of DM patients with DR (polysulfidomics) [11]. Interestingly, one of their findings was that it is possible for clinicians to infer the vitreous levels of these compounds based on their aqueous levels. However, different targeted metabolites, as well as a dissimilar study group (patients undergoing pars plana vitrectomy due to late complications of proliferative DR vs. patients with other retinal diseases, as opposed to our group of diabetic patients without any retinal disease), prevent a direct comparison to our work. Wang et al. utilized a similar study group but a diverse analytical approach (untargeted GC-TOFMS) [12]. Of note, in their study, three significantly perturbed pathways in the AH of DR patients were glycolysis or gluconeogenesis, galactose metabolism, and ascorbate–aldarate metabolism. None of the eight statistically significantly disturbed metabolites found in AH by Wang et al. coincides with the ones indicated by our research. As previously stated, this might be explained by underlying differences in early pathogenesis vs. late complications of developed DR, as well as divergent analytical approaches. Jin et al. used a somewhat different method, incorporating AH from DM, DR, and healthy control patients and utilizing an NMR-based approach [13]. Among the identified perturbed metabolites, the levels of lactate and succinate substantially decreased, and their positive correlation was observed in DR samples, which was lacking in the DM group and control group. The alanine, aspartate, and glutamate metabolism pathways were the most impactfully disturbed in DR as compared to DM. Chu et al. evaluated the AH of DM patients with and without diabetic macular edema [14]. Numerous metabolites associated with oxidative stress, hyperglycemia, inflammation, and microvascular damage were identified, among others.

One of the limitations of our research is that only selected metabolites can be measured with the AbsoluteIDQ^®^ p180 kit. However, development and more widespread use of even more wide-ranging commercial kits might mitigate this issue in the near future. The associated reduction in time necessary for metabolomics analyses might increase the number of potential applications both in the scientific and clinical fields.

## 4. Materials and Methods

### 4.1. Study Design

#### Study Participants and Sample Collection

The study included AH and serum samples from 36 diabetic patients and 36 controls (matched for sex, age, BMI, and axial length of the eye) undergoing cataract surgery in the Ophthalmology Department of the Medical University of Bialystok, Poland, from 21 January 2021 to 2 December 2021. Information on diabetes, as well as other comorbidities and prescription medicine used by the patients, was procured from a written certificate from a leading physician or self-reported in cases where such a certificate was not provided. Exclusion criteria from the study were as follows: the presence of concomitant ocular disorders (including diabetic retinopathy, excluding cataracts) and/or a history of ocular surgery or trauma.

All patients underwent a standard preoperative procedure. Venous blood (2 samples of 2.7 mL) was obtained after admission and centrifuged; plasma was gathered from the first sample for glucose metabolism assessment (with sodium fluoride as a glycolysis inhibitor) and, from the second sample, the serum was separated, frozen, and then stored at −80 °C until the metabolomic analysis. Twenty minutes before the surgery, the following were administered topically: proxymetacaine hydrochloride, tropicamide, phenylephrine, levofloxacin, diclofenac, and timolol. One hour before the surgery, patients received hydroxyzine orally for mild sedation. Standard disinfection with ophthalmic povidone-iodine 5% was performed 2 min before beginning the surgery. Before the cataract extraction, the anterior chamber of the eye was punctured using a 30 G needle; approximately 50–100 μL of AH was aspirated, transferred to Eppendorf tubes (Eppendorf, Hamburg, Germany), frozen, and stored at −80 °C until the analysis. The surgeries were performed in the morning, between 8 am and 12 pm.

### 4.2. Glucose Metabolism Assessment

Patients were investigated for anthropometric measures; multiple parameters of glucose metabolism, such as insulin and C-peptide concentrations, were evaluated using the electrochemiluminescence (ECLIA) method (07027559190, 07027168190, Cobas e411, Roche, Mannheim, Germany). Concentrations of plasma glucose were measured by hexokinase enzymatic colorimetric assay (04657527190, Cobas c111, Roche Diagnostics Ltd., Risch-Rotkreuz, Switzerland).

### 4.3. Metabolomic Analysis

Targeted metabolomic analysis of AH samples was performed using liquid chromatography coupled with tandem mass spectrometry (6470 LC-MS/MS, Agilent Technologies, Santa Clara, CA, USA) using the methodology and reagents included in the AbsoluteIDQ^®^ p180 kit (Biocrates Life Sciences AG, Innsbruck, Austria). This commercially available kit allows the quantitative measurement of 188 metabolites: 21 amino acids, 22 biogenic amines, 40 acylcarnitines, 14 lysophosphatidylcholines, 76 phosphatidylcholines, 15 sphingolipids, and the sum of all the hexoses. Sample preparation was performed according to the kit’s user manual, which has already been described in the literature [27,28], with minor modifications. A standard 10 µL of serum sample was used, as recommended by the manufacturer for plasma/serum. Because the AbsoluteIDQ^®^ p180 kit was not standardized by the manufacturer for AH, internal pre-study viability tests were performed (manuscript pending review as for date of this publication). The AH sample volume used for the analysis was optimized, and 30 µL of AH sample was used.

Spectral data processing and quantification were performed with MetIDQ software (version Oxygen-DB110-2893-0276, Biocrates, Life Science AG, Innsbruck, Austria). The performance of the analytical assay was evaluated by analyzing quality control (QC) samples at three concentration levels, whereas the middle level, QC2, was injected three times. Data below the limit of detection (LOD) were treated as missing, and filtering was performed to retain metabolites detected in at least 80% of the samples. After normalizing the data based on the QC samples, metabolites with a coefficient of variation (CV) higher than 30% in the QC samples were excluded. Subsequently, missing values were replaced with concentrations obtained based on the calibration curves but located on the calibration curve below the lowest or above the highest concentration point. All AH concentrations were divided by three to consider the higher sample volume used as compared to serum. Finally, a data matrix consisting of concentrations of metabolites was forwarded for statistical analyses.

### 4.4. Statistical Analysis

Statistical analyses were performed to identify metabolites in serum and AH differentiating diabetic and nondiabetic patients. To select statistically significant metabolic features, depending on the normality of data distribution (assessed by the Shapiro–Wilk test), a *t*-test or Mann–Whitney U test was performed. The level of statistical significance was set at 95% (*p* < 0.05). Obtained *p*-values were corrected by Benjamini–Hochberg false discovery rate (FDR), but as the study is exploratory in nature, we show metabolites significant before the correction together with corrected *p*-values. Spearman’s rank correlation was calculated for each metabolite simultaneously detected in AH and serum. Univariate statistical analyses and correlations were performed in MATLAB R2015a. SIMCA−P + 13.0.3.0 (Umetrics, Umeå, Sweden) was used for multivariate analysis. The variables were selected on a univariate basis; therefore, the models are for illustrative purposes only.

### 4.5. Pathway Analysis

Pathway analysis was conducted to identify significantly associated metabolic shifts. The pathway analysis module of the MetaboAnalyst version 5.0 toolbox was utilized to perform pathway enrichment analysis on the endogenous metabolites previously selected by univariate statistics for serum and AH, separately, using the KEGG database, Pareto scaling, no additional normalization, global test, and relative betweenness centrality.

## 5. Conclusions

The quantitative, targeted nature of our work allows our results to be more consistently compared to possible future studies adhering to similar methodology. We identified several metabolites dysregulated at the preclinical stages of diabetic retinopathy in the AH and serum of DM patients and explored the significance of these findings in relation to recent scientific discoveries. The targeted and quantitative nature of our results might indeed generate clinical value in the future. As for now, however, we are reluctant to indicate specific recommendations, as this would require further research.

## Figures and Tables

**Table 1:** Baseline patients’ characteristics.

Characteristics	DM	Control	*p*-Value
Number of patients	36	36	
Sex, female, *n* (%)	22 (61)	20 (56)	0.4
Age, mean ± SD (years)	68.69 ± 10.08	70.56 ± 7.63	0.4
BMI, mean ± SD	29.23 ± 5.98	30.05 ± 4.88	0.5
AXL, mean ± SD (mm)	23.78 ± 1.12	23.54 ± 1.10	0.4
Glu, median (IQR) (mg/dL)	133 (50)	110 (23)	0.03
INS, median (IQR) (µU/mL)	15.76 (18.83)	19.99 (13.15)	0.2
C-peptide, median (IQR) (ng/mL)	2.60 (2.39)	4.33 (1.93)	0.02

BMI, body mass index; AXL, axial length of the eye; IQR, interquartile range; Glu, fasting glucose level; INS, insulin.

**Table 2:** Metabolites in serum differentiating diabetic and nondiabetic patients.

Metabolites in Serum	*p*-Value	pBH	Average Concentration DM (µM)	Average Concentration Control (µM)	Change
PC ae C36:4	0.00004	0.005	10,699.2	15,457.7	−30.8
PC ae C34:2	0.0006	0.03	6181.1	7958.9	−22.3
PC ae C36:3	0.0004	0.03	3959.8	5216.0	−24.1
PC ae C38:4	0.002	0.05	7859.8	9552.0	−17.7
PC ae C38:5	0.002	0.06	11,756.7	14,496.1	−18.9
Tetradecenoylcarnitine	0.009	0.1	40.1	50.4	−20.3
Hexadecanoylcarnitine	0.007	0.1	50.8	63.4	−19.9
Citrulline	0.009	0.1	3878.9	6068.8	−36.1
PC aa C40:4	0.01	0.2	1862.2	2368.0	−21.4
Carnitine	0.01	0.2	5575.7	6537.9	−14.7
Arginine	0.02	0.2	15,544.1	18,524.8	−16.1
PC aa C38:3	0.02	0.2	33,204.1	40,364.0	−17.7
Octadecanoylcarnitine	0.01	0.2	23.1	28.7	−19.6
PC ae C36:5	0.03	0.2	7439.4	9239.8	−19.5
SM C20:2	0.02	0.2	233.2	293.1	−20.5
lysoPC a C18:0	0.03	0.2	9027.2	10,725.3	−15.8
Decanoylcarnitine	0.02	0.2	57.5	90.1	−36.1
PC ae C34:3	0.04	0.3	3825.6	4667.4	−18.0
PC ae C40:4	0.04	0.3	1236.4	1395.3	−11.4
Threonine	0.05	0.3	13,506.6	15,304.5	−11.7

pBH, *p*-value after Benjamini–Hochberg correction.

**Table 3:** Metabolites in aqueous humor differentiating diabetic and nondiabetic patients.

Metabolites in AH	*p*-Value	pBH	Average Concentration DM (µM)	Average Concentration Control (µM)	Change
Arginine	0.008	0.06	15,381.3	17,997.5	−14.5
Isoleucine	0.01	0.06	11,833.6	9650.9	22.6
Leucine	0.008	0.06	27,394.7	22,406.2	22.3
Valine	0.008	0.06	36,702.9	31,379.4	17.0
Alanine	0.008	0.08	37,289.7	32,079.7	16.2
Sugars	0.007	0.08	1,176,402.0	756,224.1	55.6
Propionylcarnitine	0.02	0.1	105.4	79.7	32.3
Lysine	0.03	0.1	31,953.8	28,384.8	12.6

AH, aqueous humor; pBH, *p*-value after Benjamini–Hochberg correction.

**Table 4:** Correlation coefficient between AH and serum. Correlation coefficients vary the most between groups in bold.

Metabolite Category	Sample Identification	DM Group		Control Group	
		Correlation Coefficient	*p*-Value	Correlation Coefficient	*p*-Value
**Acylcarnitines**	Carnitine	0.52	0.001	0.55	0.0005
	Acetylcarnitine	0.28	0.01	0.20	0.2
	Propionylcarnitine	0.60	0.0001	0.53	0.001
	**Butyrylcarnitine**	**0.25**	**0.1**	**0.66**	**<0.0001**
	Valerylcarnitine	0.29	0.09	0.41	0.01
Aminoacids	Alanine	0.50	0.002	0.52	0.001
	Arginine	0.38	0.02	0.47	0.004
	Asparagine	0.65	<0.0001	0.59	0.0001
	Citrulline	0.84	<0.0001	0.74	<0.0001
	Glutamine	0.39	0.02	0.61	0.0001
	**Glutamate**	**0.35**	**0.04**	**−0.04**	**0.8**
	Glycine	0.47	0.004	0.25	0.2
	Histidine	0.54	0.0007	0.34	0.04
	Isoleucine	0.54	0.0006	0.55	0.0005
	Leucine	0.45	0.006	0.45	0.006
	Lysine	0.42	0.01	0.41	0.01
	Methionine	0.54	0.0006	0.30	0.08
	Ornithine	0.63	<0.0001	0.56	0.0004
	Phenylalanine	0.47	0.004	0.37	0.03
	Proline	0.62	0.0001	0.57	0.0003
	Serine	0.61	0.0001	0.52	0.001
	Threonine	0.72	<0.0001	0.80	<0.0001
	Tryptophan	0.39	0.02	0.28	0.09
	Tyrosine	0.63	<0.0001	0.39	0.02
	Valine	0.54	0.0007	0.53	0.001
Biogenic Amines	**alpha-Aminoadipic acid**	**0.55**	**0.0005**	**0.18**	**0.3**
	Creatinine	0.88	<0.0001	0.72	<0.0001
	Kynurenine	0.59	0.0002	0.50	0.002
	trans-4-Hydroxyproline	0.83	<0.0001	0.86	<0.0001
	Taurine	0.06	0.7	0.09	0.6
Glycerophospholipids	**PC aa C34:1**	**0.07**	**0.7**	**0.37**	**0.03**
	**PC aa C38:4**	**0.08**	**0.6**	**0.47**	**0.003**
Sphingolipids	**SM C16:1**	**0.29**	**0.08**	**−0.11**	**0.5**
	SM C18:1	−0.03	0.8	−0.02	0.9
Sugars	Hexoses	0.67	<0.0001	0.65	<0.0001

**Table 5:** Numerical values obtained from pathway analysis performed for serum metabolites significantly discriminating between diabetic and nondiabetic patients.

Pathway Name	Match Status	*p*	−log(*p*)	Holm *p*	FDR	Impact
Arginine biosynthesis	2/14	0.0011	2.95	0.094	0.094	0.30
2. Aminoacyl-tRNA biosynthesis	2/48	0.013	1.89	1	0.4	0
3. D-Arginine and D-ornithine metabolism	1/4	0.015	1.81	1	0.4	0
4. Linoleic acid metabolism	1/5	0.019	1.71	1	0.4	0
5. Valine, leucine, and isoleucine biosynthesis	1/8	0.03	1.51	1	0.51	0
6. alpha-Linolenic acid metabolism	1/13	0.049	1.3	1	0.69	0
7. Glycine, serine, and threonine metabolism	1/33	0.12	0.92	1	1	0
8. Arachidonic acid metabolism	1/36	0.13	0.88	1	1	0
9. Glycerophospholipid metabolism	1/36	0.13	0.88	1	1	0.095
10. Arginine and proline metabolism	1/38	0.14	0.86	1	1	0.058
11. Fatty acid degradation	1/39	0.14	0.85	1	1	0

**Table 6:** Numerical values obtained from pathway analysis performed for AH metabolites significantly discriminating between diabetic and nondiabetic patients.

Pathway Name	Match Status	*p*	−log(*p*)	Holm *p*	FDR	Impact
Aminoacyl-tRNA biosynthesis	6/48	<0.0001	6.58	<0.0001	<0.0001	0
2. Valine, leucine, and isoleucine biosynthesis	3/8	<0.0001	4.83	0.0012	0.0006	0
3. Valine, leucine, and isoleucine degradation	3/40	0.0023	2.64	0.19	0.064	0
4. Galactose metabolism	2/27	0.015	1.84	1	0.31	0.052
5. Amino sugar and nucleotide sugar metabolism	2/37	0.027	1.57	1	0.39	0
6. D-Arginine and D-ornithine metabolism	1/4	0.028	1.55	1	0.39	0
7. Ascorbate and aldarate metabolism	1/8	0.056	1.26	1	0.67	0
8. Biotin metabolism	1/10	0.069	1.16	1	0.72	0
9. Arginine biosynthesis	1/14	0.095	1.02	1	0.89	0.076
10. Pantothenate and CoA biosynthesis	1/19	0.13	0.9	1	1	0
11. Selenocompound metabolism	1/20	0.13	0.87	1	1	0
12. Lysine degradation	1/25	0.16	0.78	1	1	0
13. Glycolysis/gluconeogenesis	1/26	0.17	0.77	1	1	0.0002
14. Alanine, aspartate, and glutamate metabolism	1/28	0.18	0.74	1	1	0
15. Phosphatidylinositol signaling system	1/28	0.18	0.74	1	1	0.037
16. Inositol phosphate metabolism	1/30	0.19	0.71	1	1	0.13
17. Arginine and proline metabolism	1/38	0.24	0.62	1	1	0.058

## Data Availability

The datasets generated and analyzed during the current study are available from the corresponding author upon reasonable request.

## Supplementary Materials

The supporting information can be downloaded at: https://www.mdpi.com/article/10.3390/ijms241612671/s1.
